# First Reported Case of Gabriele-de Vries Syndrome with Spinal Dysraphism

**DOI:** 10.3390/children10040623

**Published:** 2023-03-26

**Authors:** Nenad Koruga, Silvija Pušeljić, Marko Babić, Mario Ćuk, Andrea Cvitković Roić, Vjenceslav Vrtarić, Anamarija Soldo Koruga, Alen Rončević, Višnja Tomac, Tatjana Rotim, Tajana Turk, Domagoj Kretić, Nora Pušeljić, Rebeka Nađ, Ivana Serdarušić

**Affiliations:** 1 Department of Neurosurgery, University Hospital Center Osijek, 31000 Osijek, Croatia; 2 Faculty of Medicine, Josip Juraj Strossmayer University of Osijek, 31000 Osijek, Croatia; 3 Department of Pediatrics, University Hospital Center Osijek, 31000 Osijek, Croatia; 4 Department of Surgery, University Hospital Center Osijek, 31000 Osijek, Croatia; 5 Department of Pediatrics, University Hospital Center Zagreb, 10000 Zagreb, Croatia; 6 School of Medicine, University of Zagreb, 10000 Zagreb, Croatia; 7 Helena Clinic for Pediatric Medicine, 10000 Zagreb, Croatia; 8 Department of Neurology, University Hospital Center Osijek, 31000 Osijek, Croatia; 9 Department of Diagnostic and Interventional Radiology, University Hospital Center Osijek, 31000 Osijek, Croatia

**Keywords:** Gabriele-de Vries syndrome, spinal dysraphism, whole-exome sequencing, YY1, Arnold-Chiari malformation

## Abstract

Gabriele-de Vries syndrome is a rare autosomal dominant genetic disease caused by de novo pathogenic variants in the *Yin Yang 1* (*YY1)* gene. Individuals with this syndrome present with multiple congenital anomalies, as well as a delay in development and intellectual disability. Herein, we report the case of a newborn male patient with a novel de novo pathogenic variant in the Guanine Nucleotide-Binding Protein, Alpha Stimulating (*GNAS*) gene, which was identified by whole-exome sequencing. Our patient suffered from a large open spinal dysraphism which was treated surgically immediately after birth. During the follow-up, facial dysmorphism, bladder and bowel incontinence, and mildly delayed motor and speech development were observed. Congenital central nervous system disorders were also confirmed radiologically. In this case report, we present our diagnostic and treatment approaches to this patient. To our knowledge, this is the first reported case of Gabriele-de Vries syndrome presenting with spinal dysraphism. Extensive genetic evaluation is the cornerstone in treatment of patients with suspected Gabriele-de Vries syndrome. However, in cases with potentially life-threatening conditions, surgery should be strongly considered.

## 1. Introduction

An extremely uncommon autosomal dominant (AD) disorder, called Gabriele-de Vries syndrome (GADEVS), is characterized by mild to profound intellectual disability, craniofacial dysmorphism, and a number of congenital anomalies. The diagnosis of GADEVS is established with genetic testing by identification of a heterozygous pathogenic variant involving the *Yin Yang 1* (*YY1)* gene or a heterozygous deletion of 14q32.2 involving only *YY1* gene. The *YY1* gene deletion or pathogenic mutation causes GADEVS (OMIM 600013) [1]. The zinc finger transcription factor YY1 is encoded by the *YY1* gene, which is found on chromosome 14q32.2. According to interaction partners, promoter context, and chromatin structure, this specific transcription factor regulates a variety of physiological activities, such as differentiation, DNA repair, autophagy, cell proliferation, and apoptosis, by either activating or inhibiting gene expression [2]. Only twelve cases of GADEVS have been described to date, and all of these patients had deletions encompassing *YY1* and other nearby genes or loss-of-function mutations in the zinc fingers of the *YY1* gene [1,3,4]. A de novo *YY1* gene pathogenic mutation or deletion causes the condition in all probands with GADEVS who have been documented to date and whose parents have undergone molecular genetic testing. Besides aforementioned neurodevelopmental and intellectual disorders, the most common clinical findings include craniofacial dysmorphisms, neurologic abnormalities and behavioral disorders, multiple congenital organ anomalies, and intrauterine growth restriction followed by a low birth weight. Herein, we describe a case of a newborn male patient with GADEVS presented with an open spina bifida of the lumbosacral region. To our knowledge, this is the first reported case of spinal dysraphism in a patient with GADEVS. Whole genome sequencing (WGS) identified one causative variant in exon 1 of the *YY1* gene. Among the other variants identified, we emphasize probably pathogenic variant of the Guanine Nucleotide-Binding Protein, Alpha Stimulating (*GNAS*) gene in a heterozygous composition, which our proband inherited from the mother (c.1360C > T, p.GIn454* with a presumed Stop Gain effect).

## 2. Case Report

**Figure 1:**
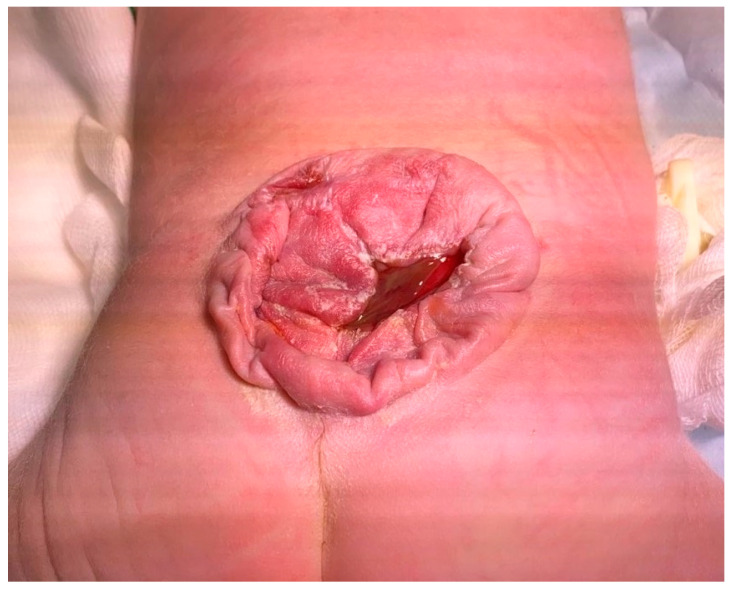
Intraoperative image of the large lumbosacral meningomyelocele.

Our patient was born from the first regularly controlled pregnancy of young and healthy parents (father was 32 and mother was 27 years old). According to their familial history, there was no evidence of consanguinity. There was no history of previous pregnancies, still births, or abortions. Parents were non-smokers and did not consume drugs or alcohol. Since the confirmation of the pregnancy in the fifth week, mother had routinely supplemented with folic acid. In addition, there was no data of familial neurological diseases or spinal dysraphism. During the pregnancy, an enlargement of the lateral ventricles, oligohydramnion, and distended bladder were observed with ultrasound. The delivery of the baby was at term with an emergency section. A full-term male child was born with a birth mass of 3720 g, Apgar score 7/8, and a large lumbosacral meningomyelocele (Figure 1).

The patient was urgently surgically treated immediately after birth. The surgery was performed by a neurosurgeon and a plastic surgeon in general anesthesia in a regular fashion according to the algorithm for an open spina bifida. A plastic surgeon performed reconstructive rotational skin flap due to a large skin defect. Eleven days after the surgery, the large fontanelle was protruded and tense. The cranial ultrasound confirmed hydrocephalus. Therefore, a ventriculo-peritoneal (VP) shunt was implanted in general anesthesia (Medtronic^®^ uni-shunt, medium pressure). Postoperative radiologic evaluation confirmed a properly placed VP drainage and a slightly wider ventricular system. Further postoperative recovery of the child before discharge was uneventful.

**Figure 2:**
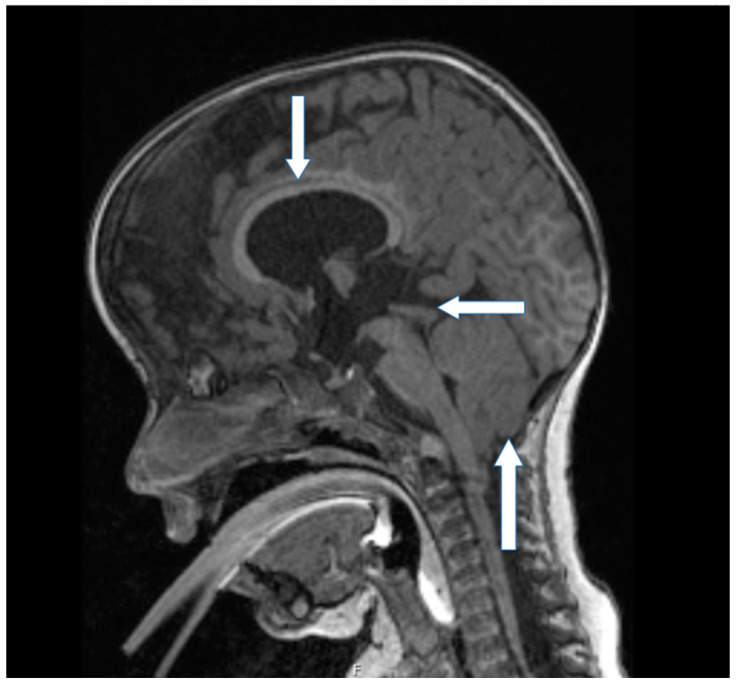
Non-enhanced sagittal MRI T1-weighted scan revealed a Chiari malformation type 2 (up arrow), hypoplasia of the corpus callosum (down arrow), and dysgenesis of the lamina tecti (left arrow).

**Figure 3:**
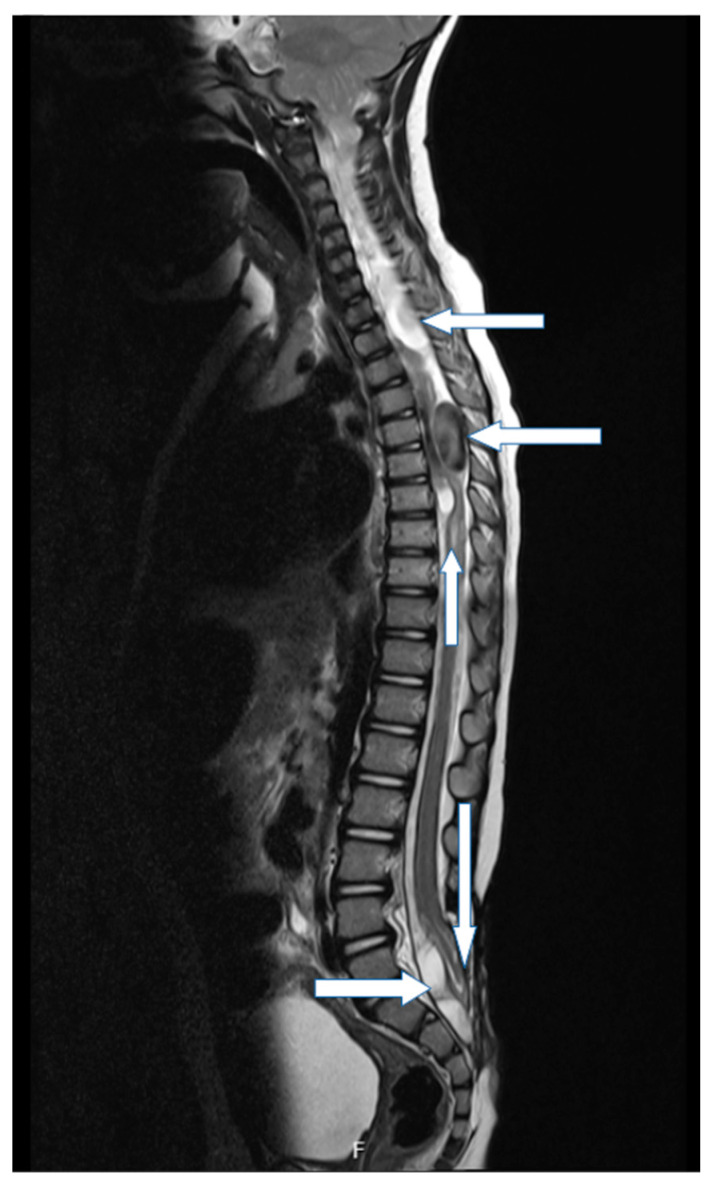
Sagittal MRI T2-weighted scan revealed spinal cysts in the thoracic (left arrows) and lumbar regions (right arrow), syringomyelia (up arrow) and tethered cord (down arrow).

The child was later followed by a pediatrician through the outpatient clinic for genetic diseases. According to its clinical evaluation, further karyotype testing was recommended to exclude potential genetic disorders. Also, the child was appropriately observed by a neurosurgeon, and a magnetic resonance imaging (MRI) of neuroaxis was performed twice per year. The postoperative MRI revealed a Chiari malformation type 2, syrinx, spinal cysts, and tethered cord (Figure 2 and Figure 3). Despite aforementioned disorders verified by the MRI, the child was observed and conservatively treated due to its proper development of fine motor and oculomotor skills, while developmental delay of gross motor skills was noted. Psychomotor development was slow, and the child underwent Vojta-therapy. At fifteen months of age the patient was able to speak a few words, walked independently, and was able to sit on his own.

At the age of two years, the psychologist evaluated our patient as an emotionally warm, cheerful, and sociable boy with proper cognitive, speech-language, and socio-emotional development. The child walked completely independently at this age. Dysmorphic features of the child include swollen eyelids, a small chin, scaphocephaly, and an elongated body. As a result of meningomyelocele, our patient later developed a neurogenic bowel and bladder. He also underwent a pediatric urologist evaluation due to a bladder dysfunction; gradually, its emptying improved.

## 3. Genetic Evaluation and Interpretation of Results

Karyotype revealed a 46, XY-normal male; DNA analysis using the chromosomal microarray analysis (CMA) revealed: arr(X,Y)x1,(1-22)x2. WGS has been conducted from the peripheral blood. It revealed one pathogenic and two probably pathogenic variants presented in Table 1.

Methodology: samples are subjected to a quality control (QC) evaluation before library construction and robotic library preparation, followed by a library QC. WGS is performed in a vendor laboratory on the Illumina NovaSeq 6000 platform. The reads from this sequencing are aligned to a reference sequence (genome build hg37). Clinical-grade nuclear genome sequencing is performed at an average coverage of 40X with a minimum depth of coverage of 35X. Over 90% of the autosomal genome is sequenced to a depth of >10X. The mitochondrial genome is sequenced to a minimum depth of 20X. 

Data analysis, detection of genome alterations, filtration, case workup, genome interpretation, and result generation are all performed by the genomic platforms, systems, pipelines, and tools at the MGB PI’s genomic unit.

Limitation: the findings reported are those identified by WGS analysis of this subject’s blood sample with potential relevance, as determined by the pipeline used herein. Some sequence alterations may have been detected that are known to have no genetic relevance, or that were judged to have insufficient evidence of genetic utility by the pipeline herein and, therefore, are not reported. This genome sequencing and the analysis herein does not detect genome rearrangements, translocations, their breakpoint in introns, and repeat expansion alterations. Detection of those alterations requires different technology or methods. Furthermore, the interpretation of variants is performed based on current understanding of the specific genes and genetic alterations. The interpretation may change over time as more information about this gene becomes available. These results should not be viewed as a substitute for diagnostic testing; results should be interpreted in the context of personal and family history and other data. Due to limitations in technology, certain regions may either not be covered or may be poorly covered, where variants cannot be confidently detected, and some variants may not have a good coverage or quality or may need an orthogonal confirmation. Only variations in genes potentially related to the proband condition are reported. In line with the American College of Medical Genetics’ (ACMG) recommendations for reporting of incidental findings in genome sequencing, pathogenic and likely pathogenic variants only in the recommended genes may be reported. All reported alterations follow the ACMG classification guidelines [4].

*GJB2*-variant detail: the c.109G > A variant is a 1 base pair substitution in the exon 2 of the *GJB2* gene that results in the protein change p.Val37lle. This variant has been observed in the gnomAD database at a population allele frequency of 0.772% (Exome) and 0.624% (Genome). It has been frequently reported in individuals affected with mild to moderate deafness. The c.109G > A has been shown to segregate with autosomal recessive deafness in families. This variant has been detected in trans with a pathogenic variant [5]. This variant has been reported in the ClinVar database (variation ID: 17023). In summary, this variant is interpreted as pathogenic. Mutations in this gene are responsible for as much as 50% of prelingual, recessive deafness [6].

*YY1*-variant detail: the c.1A > C variant is a substitution in the exon 1 of the *YY1* gene. It affects the methionine residue at the start codon, and it is anticipated that the resulting protein product will be missing or distorted. Function loss is a recognized disease mechanism (pLI = 0.99, o/e = 0 10. 0.2]). This variant has not been reported in the gnomAD database. This variant has not been characterized in the literature. In summary, this variant is interpreted as likely pathogenic. 

The protein is involved in both the activation and repression of a wide range of promoters. Histone modification is implicated in the function of *YY1*, because *YY1* may direct histone deacetylases and histone acetyltransferases to a promoter in order to activate or repress the promoter [7].

*GNAS*-variant detail: the c.1360C > T variant is a nonsense substitution in the *GNAS* gene that results in a premature stop codon. Due to the location of the premature termination codon, the resulting mRNA is expected to undergo degradation that in turn can result in the absence of the protein product from that allele. Loss of function is an established mechanism of disease [8]. This variant has not been reported in the gnomAD database. This variant has not been characterized in the literature. In summary, this variant is interpreted as likely pathogenic. An extremely intricate imprinted expression pattern exists at this locus. From four different promoters and 5′ exons, it produces maternally, paternally, and biallelically expressed transcripts. The antisense transcript and one of the transcripts generated from this locus are paternally expressed noncoding RNAs that may control imprinting in this area. A second overlapping ORF that encodes the structurally-unrelated protein Alex is also present in one of the transcripts [9]. The stimulatory G-protein alpha subunit is a crucial component of the traditional signal transduction pathway connecting receptor-ligand interactions with the activation of adenylyl cyclase and a variety of cellular responses. Alternative splicing of downstream exons is also observed, resulting in various forms of the stimulatory G-protein alpha subunit. For this gene, many transcript variants have been discovered that encode various isoforms. Pseudohypoparathyroidism type 1a, type 1b, Albright hereditary osteodystrophy, pseudohypoparathyroidism, McCune-Albright syndrome, progressive osseus heteroplasia, polyostotic fibrous dysplasia of bone, and various types of pituitary tumors are all caused by mutations in this gene [10].

## 4. Discussion

In this article, we presented the first case of GADEVS with a large lumbosacral meningomyelocele. GADEVS was first described in 2017 by Gabriele et al. [1], and in the following years more cases were reported. It is caused by pathogenic variants in *YY1* which is located in the telomeric region of chromosome 14 at band q32.2. GADEVS is an autosomal dominant neurodevelopmental disorder characterized by a delay in psychomotor development, variable cognitive deficits, often with behavioral problems, feeding problems, some movement disorders, and dysmorphic facial features [11]. Other developmental anomalies may also be present.

Our proband was born with a large congenital lumbosacral meningomyelocele. In addition, dysgenetic changes of the lamina tecti, hypoplasia of the corpus callosum, Chiari malformation type 2, postoperative syringomyelia, and tethered cord have been observed with radiological imaging. 

A certain dispute existed regarding the Chiari type in our proband. At a first glance, radiological assessment was Chiari type 3 due to the existence of a minor occipital encephalocele—although, the appearance of the posterior fossa content was interpreted as Chiari type 2; the latter type is characterized by protrusion of vermis, cerebellar tonsils and the brainstem through the foramen magnum and is usually seen in patients with meningomyelocele. Compared to other types of Chiari malformations, type 3 is the least common to be observed in clinical practice. It is distinguished by a low occipital and high cervical encephalocele, as well as the herniation of the cerebellum and/or brainstem, occipital lobe, and fourth ventricle from the posterior fossa.

Facial dysmorphism was presented in our proband, and according to the review article by Khamirani et al., facial dysmorphism was presented in all described GADEVS patients [12,13,14]. No defects of other organ systems have been verified in our proband. Under these circumstances, the child was primarily treated surgically at the day of birth. Lumbosacral meningomyelocele was meticulously closed and the right-sided VP drainage was later implanted. 

The genetic workup started with karyotype testing which ruled out chromosomal abnormalities. Concomitant CMA also revealed no abnormalities. WGS was performed and a causative variant in the exon 1 of the *YY1* gene was identified by high-precision sequencing of the child’s genome. It is a point mutation that changes the start codon and, as a result, the protein product is not created—i.e., its function is impaired which is a known cause of the disease. This variant has not yet been described in the literature, but it is classified as probably pathogenic and, in our patient, it arose de novo. The *YY1* gene creates an entry for the transcriptional repressor protein YY1 (TYY1). TYY1 is a ubiquitous transcription factor that belongs to the GLI-Kruppel class of zinc finger proteins and is involved in the repression and activation of several types of promoters. Besides this probably pathogenic variant, WGS identified two more variants. The clinical significance of the GNAS gene mutation and its contribution to the clinical presentation needs further assessment, particularly periodic follow-up of the child and his mother. Symptoms can be clinically developed from childhood to adulthood and most frequently include muscle spasms, cataracts, dental issues, or polyostic fibrous dysplasia of the, but the phenotype can be variable. The gene variant *GJB2* c.109G:&gt;A is found in people with mild to moderate deafness, but the disease is inherited according to an autosomal recessive model—our proband is the carrier of this variant. Interpretation of WGS findings correlated with a clinical and radiologic presentation of symptoms—i.e., the identified genotype corresponded to the phenotype of the patient—GADEVS, OMIM #617557 [11].

Current experience and knowledge about this syndrome indicate a variable clinical course and presentation, depending on the type of mutation. Reviewing the literature, we did not find a patient with GADEVS and developmental meningomyelocele previously described. Only several patients with delayed myelination, frontal gliosis, ventriculomegaly, and white matter abnormalities were observed. Authors identified de novo likely pathogenic mutation in *YY1* gene in a patient exhibiting clinical features and phenotype characteristics of GADEVS. According to the ACMG standards and guidelines, the variation is classified as likely pathogenic. This is the first report of a patient with a *YY1*-associated syndrome in the republic of Croatia, and is also the first-described GADEVS case in the literature with meningomyelocele. Further clarification of molecular mechanism is needed, and new studies should be strongly considered. Conservative treatment and supportive care remain the only viable possibilities. Further clinical monitoring of our patient includes a multidisciplinary approach.

## Figures and Tables

**Table 1:** Summary table of the whole-genome sequencing–variants c.1A > C and c.1360C > T have not been characterized in the literature.

Gene	cDNA	Protein	Zygosity	Inheritance	Classification
GJB2	c.109G > A	p.Val37lle	Heterozygous	Maternal	Pathogenic
YY1	c.1A > C	p.Met1?	Heterozygous	De novo	Likely pathogenic
GNAS	c.1360C > T	p.Gln454*	Heterozygous	Maternal	Likely Pathogenic

## Data Availability

Not applicable.
